# Hemophagocytic Lymphohistiocytosis in Dermatomyositis

**DOI:** 10.4274/balkanmedj.2018.1339

**Published:** 2019-01-01

**Authors:** Hsien-Tzung Liao, Ching-Fen Yang, Chang-Youh Tsai

**Affiliations:** 1 Department of Internal Medicine, Division of Allergy, Immunology and Rheumatology, Taipei Veterans General Hospital, Taipei, Taiwan; 2 National Yang-Ming University, School of Medicine, College of Medicine, Taipei, Taiwan; 3 Department of Pathology and Laboratory Medicine, Taipei Veterans General Hospital, Taipei, Taiwan

A 58-year-old Asian woman suffered from a high-spiking fever (39.5-40°C), general malaise, and a body weight loss of 6 kg over several months. She presented to our clinic with progressive proximal girdle weakness, scaly erythematous papules on the extensor sides and knuckles of the bilateral metacarpophalangeal/proximal interphalangeal and elbow joints (Gottron’s papules, Figure 1a), as well as a heliotrope sign with a purplish halo around the eyes. This constellation of presentations was initially thought to favor a diagnosis of dermatomyositis because there was also electromyographic evidence of spontaneous fibrillations and a low-amplitude, polyphasic motor unit potential of short duration (1). Laboratory data revealed persistent leukopenia (1930-2300/uL) and high serum concentrations of lactate dehydrogenase (534 U/L) and ferritin (2647.8 ng/mL). To further explore the possibility of occult infections or a malignancy, a bone marrow examination was performed. Significantly increased histiocytes (Figure 1b, highlighted by CD68 immunohistochemistry stain, brown color, arrows) with hemophagocytosis, macrophages engulfing lymphocytes, and cell debris in the cytoplasm (Figure 1c, Liu stain, arrow) were demonstrated. Hemophagocytic lymphohistiocytosis associated with dermatomyositis was thus diagnosed. After aggressive treatment with systemic steroids (intravenous methylprednisolone 1 g/day for 3 days followed by a maintenance dose of 2 mg/kg/day orally) and cyclosporine-A (2.5 mg/kg/day), the hemophagocytic lymphohistiocytosis and dermatomyositis were well controlled. Then, written informed consent was obtained from the patient because of robustness.

Hemophagocytic lymphohistiocytosis, also called macrophage activated syndrome or hemophagocytic syndrome, is a life-threatening disease with a severe inflammatory reaction caused by fulminant and uncontrolled activation of lymphocytes, macrophages, and proinflammatory cytokines (2). Hemophagocytic lymphohistiocytosis always brings catastrophic damage to the hematologic and immunologic systems and it is considered a type of cytokine storm. When encountering such a patient with an autoimmune disease such as dermatomyositis and presentations including persistent leukopenia, ferocious fever, and hyper-ferritinemia, physicians should keep in mind that besides infections and malignant neoplasm, hemophagocytic lymphohistiocytosis is another possible diagnosis.

## Figures and Tables

**Figure 1:**
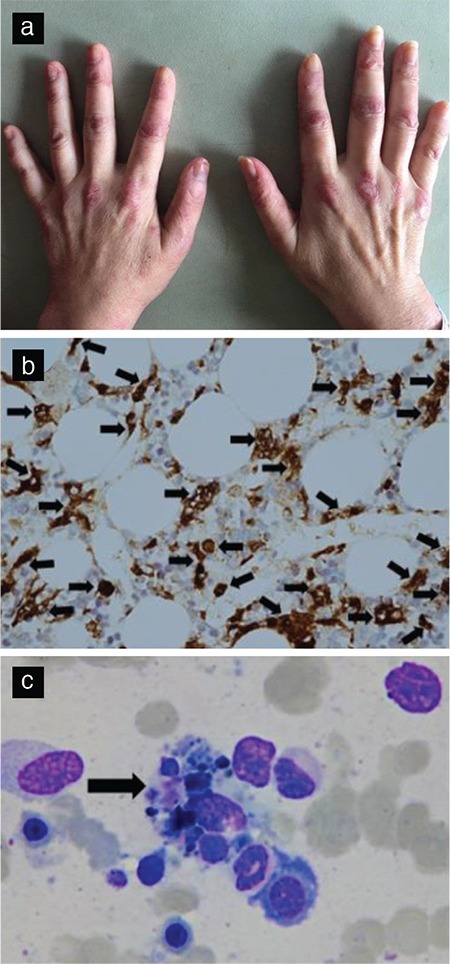
Gottron’s papules over the knuckle area of the hands in dermatomyositis (a). Increased histiocytes (highlighted by CD68 immunohistochemistry staining, brown color, 400× magnification, arrows) (b) with hemophagocytosis, a macrophage engulfing lymphocytes, and cell debris in the cytoplasm (Liu stain, 400× magnification, arrow) in a bone marrow biopsy (c).
